# Radiologic Characteristics and Clinical Correlates of Quadrigeminal Cistern Arachnoid Cysts: A Retrospective Study

**DOI:** 10.7759/cureus.102371

**Published:** 2026-01-27

**Authors:** Fatemeh Azizi, Emrah Karatay, Abdulkadir Eren, Emre Karacay

**Affiliations:** 1 Radiology, Sultan II Abdulhamid Han Training and Research Hospital, Istanbul, TUR; 2 Radiology, Medipol Mega University Hospital, Istanbul, TUR; 3 Radiology, Ministry of Health Susurluk State Hospital, Balıkesir, TUR

**Keywords:** arachnoid cyst, evans index, mega cisterna magna, quadrigeminal cistern, vertebral artery

## Abstract

Introduction

Quadrigeminal cistern arachnoid cysts (QACs) represent a rare subgroup of arachnoid cysts. Patients may present with headache, visual disturbances, gait imbalance, or nausea, typically secondary to obstructive hydrocephalus. Hydrocephalus is frequently assessed using the Evans Index (EI). Vertebral artery hypoplasia (VAH) is generally defined as a decrease in the diameter of the vertebral artery. It has been associated with posterior circulation ischemia, vertebrobasilar insufficiency (VBI), vertigo, and headache. However, the relationship between QAC-related mass effect and vascular variants such as VAH has not been systematically evaluated. Mega cisterna magna (MCM) represents another posterior fossa cerebrospinal fluid variant relevant to the differential diagnosis.

Objective

To evaluate the relationship between QAC dimensions, EI, vertebral artery diameters, and clinical symptoms, and to assess the coexistence of MCM.

Methods

Seventy-three patients (36 females, 37 males; mean age 52.4 ± 19.0 years) with radiologically confirmed QAC were retrospectively reviewed. Clinical symptoms, including headache and VBI, were recorded. Imaging parameters included QAC anteroposterior (AP) diameter, quadrigeminal cistern diameter, EI, vertebral artery diameters, and the presence of MCM. Multivariable logistic regression analysis was performed to identify imaging predictors of clinical symptoms (odds ratio (OR) and confidence interval (CI)).

Results

Headache was present in 67.1% of patients, VBI in 31.5%, and MCM in 21.9%. Mean QAC AP diameter was 16.9 ± 4.8 mm, quadrigeminal cistern diameter 23.1 ± 5.2 mm, and EI 0.24 ± 0.08. Mean right and left vertebral artery diameters were 2.46 ± 0.83 mm and 2.36 ± 0.73 mm, respectively. Headache showed no independent association with any imaging parameter (p-value > 0.05). VBI was significantly associated with a smaller left vertebral artery diameter (OR 0.32; 95% CI 0.16-0.64; p-value = 0.001).

Conclusion

In patients with QAC, headache did not correlate with radiologic measurements, whereas VBI was strongly associated with reduced vertebral artery diameter, particularly on the left side. These findings highlight the importance of incorporating vascular assessment when evaluating symptomatic QAC patients.

## Introduction

Arachnoid cysts are benign, cerebrospinal fluid (CSF)-filled lesions that account for approximately 1% of all intracranial space-occupying masses. Quadrigeminal cistern arachnoid cysts (QACs) represent a rare subset, comprising 5-18% of all arachnoid cysts [1,2]. Because the quadrigeminal cistern is located posterior to the tectal plate and adjacent to the aqueduct of Sylvius, cysts in this region may exert mass effect on the aqueduct, brainstem, and surrounding venous structures [3]. Patients may present with headache, visual disturbances, gait imbalance, or nausea, typically secondary to obstructive hydrocephalus [1-5].

Hydrocephalus is frequently assessed using the Evans Index (EI), defined as the ratio of maximal frontal horn width to inner cranial diameter at the same axial level. An EI greater than 0.30 is commonly used to indicate ventriculomegaly; however, age-related changes and borderline values may limit its diagnostic specificity [6]. Objective quantification of ventricular size is therefore important when evaluating the clinical impact of QAC [6,7].

Beyond CSF dynamics, alterations in posterior circulation hemodynamics may also contribute to symptoms [8,9]. Vertebral artery hypoplasia (VAH) is commonly defined as an arterial diameter <2.0 mm. It has been associated with posterior circulation ischemia, vertebrobasilar insufficiency (VBI), vertigo, and headache [8-10]. VBI encompasses a range of symptoms, including dizziness, diplopia, imbalance, and drop attacks, and reflects compromised vertebrobasilar flow reserve [11]. However, the relationship between QAC-related mass effect and vascular variants such as VAH has not been systematically evaluated.

Mega cisterna magna (MCM) represents another posterior fossa CSF variant relevant to the differential diagnosis. MCM is generally considered an incidental imaging finding, characterized by enlargement of the cisterna magna without associated hydrocephalus or vermian hypoplasia [12,13]. Distinguishing MCM from arachnoid cysts is essential because arachnoid cysts may compress adjacent structures, whereas MCM is usually asymptomatic. The coexistence of MCM and QAC, and its potential clinical implications, remain insufficiently explored [14].

Given these considerations, we hypothesized that both ventricular enlargement and vertebrobasilar vascular variants may influence symptom burden in patients with QAC. Accordingly, the objective of this study was to investigate the relationships between QAC size, EI, vertebral artery diameters, quadrigeminal cistern dimensions, and the presence of headache and VBI symptoms. We also evaluated the coexistence of MCM to more comprehensively characterize posterior fossa CSF space variants in this patient population.

## Materials and methods

Ethics approval

This study was conducted in accordance with the Declaration of Helsinki. Approval was obtained from the İstanbul Medipol University Institutional Review Board (IRB no: E-10840098-202.3.02-5773). The requirement for informed consent was waived because of the retrospective design and anonymization of patient data.

Study design and population

This retrospective observational study included 73 patients with QACs diagnosed by neuroimaging at the Department of Radiology between January 2018 and December 2024.

The cases with arachnoid cysts radiologically confirmed to be located in the quadrigeminal cistern by computed tomography (CT) or magnetic resonance imaging (MRI), and for whom archival records of clinical findings were available, were included in the study. Patients with other posterior fossa malformations (e.g., Dandy-Walker malformation), a history of severe head trauma, previous neurosurgical intervention, and inadequate imaging quality for measurement were excluded from the study.

Data collection

Clinical information about the cases was extracted from electronic medical records, including headache (present/absent), demographic data (age and gender), symptoms of VBI defined as dizziness, vertigo (+/-), balance disorder, double vision, or falls according to established neurological criteria [9].

Imaging protocol and analysis

**Figure 1:**
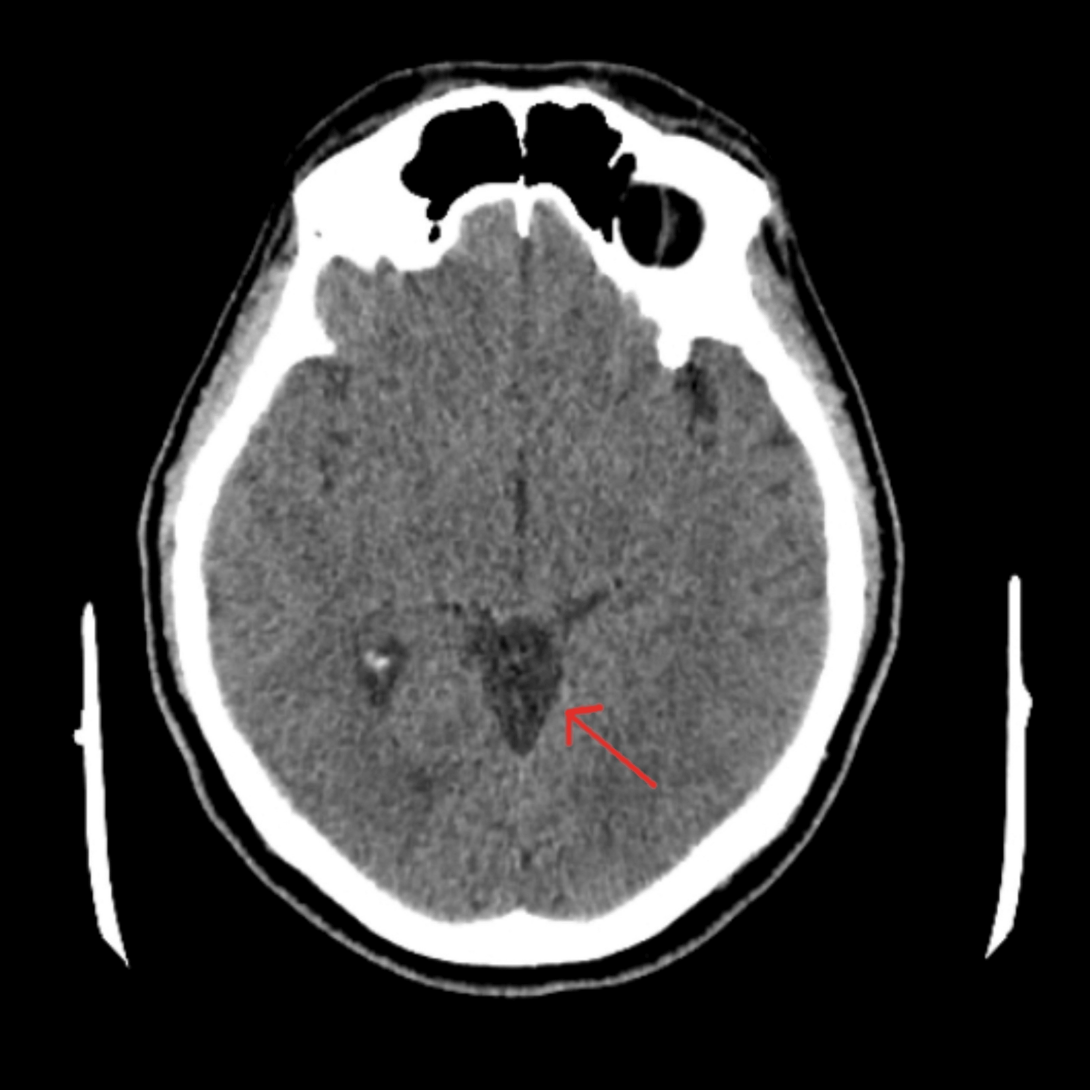
Axial non-contrast CT image demonstrating a well-defined, hypodense cerebrospinal fluid-attenuation lesion in the quadrigeminal cistern, consistent with an arachnoid cyst (arrow).

**Figure 2:**
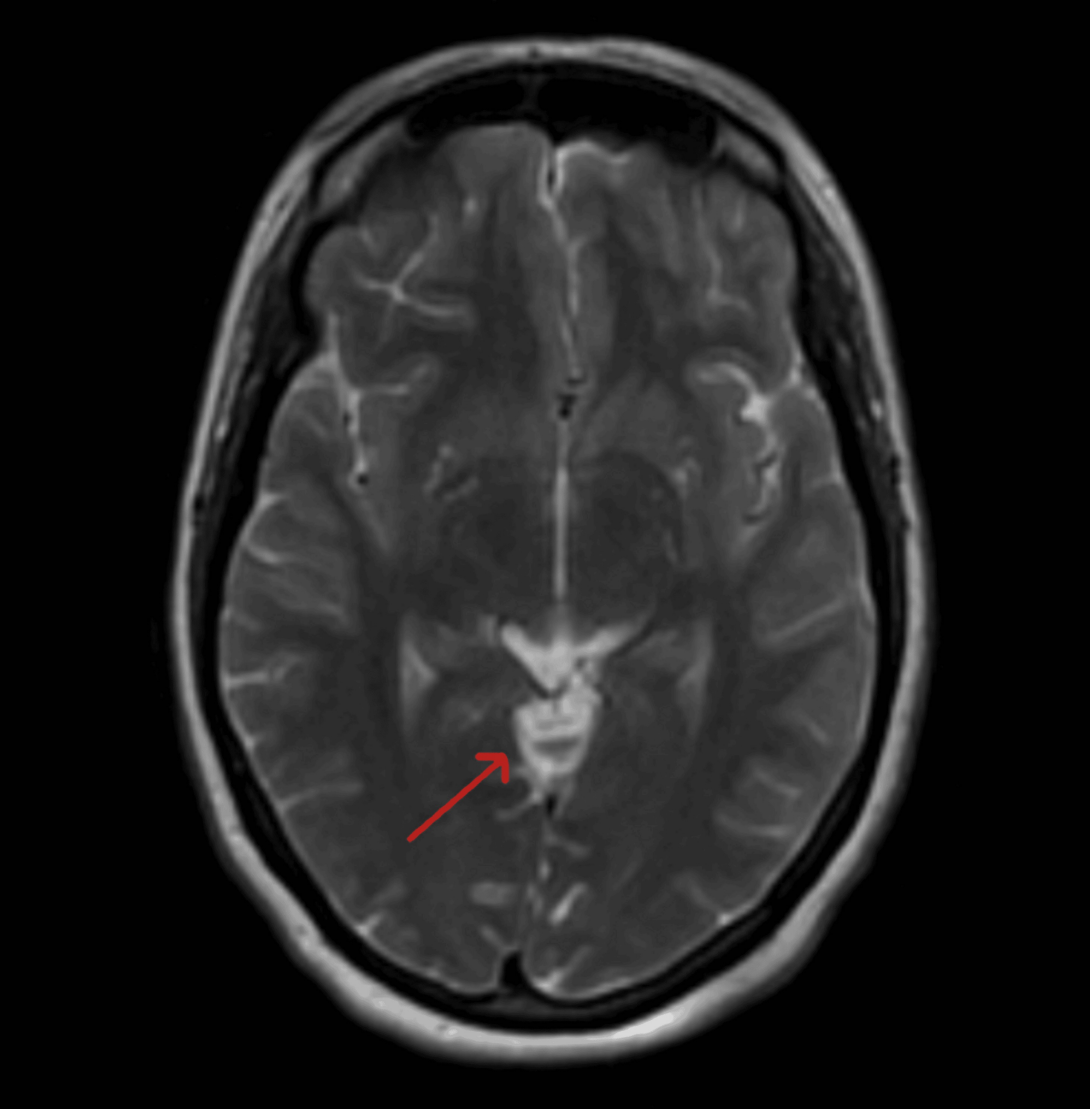
Axial T2-weighted MRI confirming a CSF-intensity lesion in the quadrigeminal cistern with typical signal characteristics of an arachnoid cyst and without associated parenchymal edema (arrow). CSF: cerebrospinal fluid

For patients with available multislice CT images, thin-slice axial posterior fossa scans with coronal and sagittal reconstructions were evaluated. For MRI, images from 1.5T (Tesla) systems with axial T1-weighted, T2-weighted, fluid-attenuated inversion recovery (FLAIR), and diffusion-weighted sequences were evaluated. This also allowed for measurements of QAC and MCM for the patients. Representative CT and MRI images of QACs are shown in Figures 1-2, respectively.

**Figure 3:**
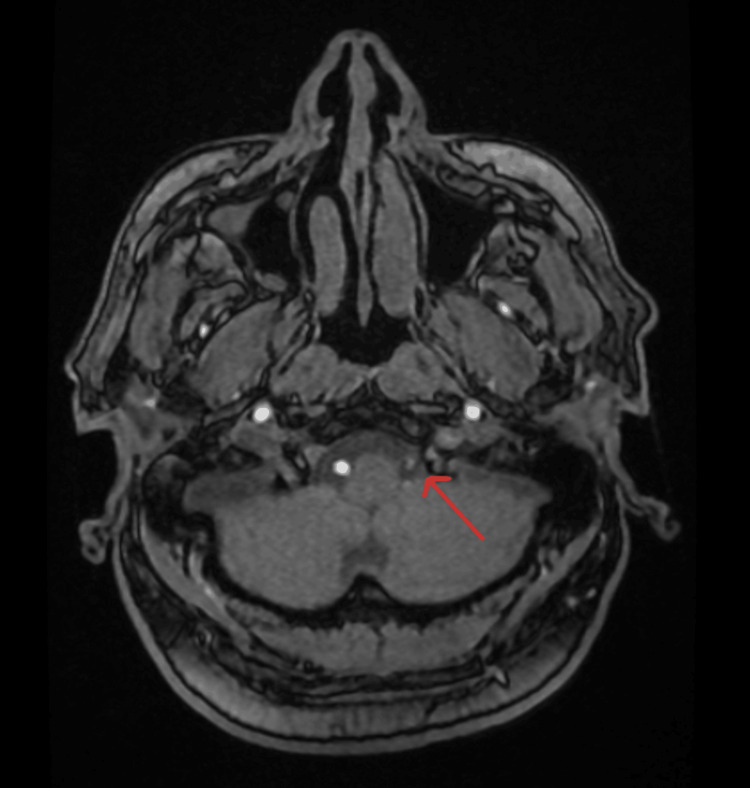
Axial T1-weighted image demonstrating measurement of the vertebral artery at the V4 segment (red arrow). The vessel caliber was recorded bilaterally on MR angiography for evaluation of vertebral artery hypoplasia and its relationship to vertebrobasilar insufficiency.

For vascular imaging, time-of-flight (TOF) and/or contrast-enhanced MR angiography (MRA) were used, as well as CT angiography (CTA) images (when available) for vertebral artery assessment. Figure 3 shows the vertebral artery measurement methodology in cranial MRA.

All images were independently reviewed by two neuroradiologists with over five years of experience; discrepancies were resolved by consensus. Interobserver agreement for continuous variables was assessed using the intraclass correlation coefficient (ICC).

QAC size was measured as the maximum anteroposterior (AP) diameter in axial images, and quadrigeminal cistern diameter was measured as the maximum CSF width behind the tectal plate at the level of the superior colliculi.

EI was calculated as the ratio of the maximum anterior horn width to the maximum internal skull diameter in the same axial section, and EI > 0.30 was considered ventriculomegaly [4,5]. Vertebral artery diameters were measured in the V4 segment on MRA, and VAH was defined as <2.0 mm [6-8]. MCM is when the AP diameter of the cisterna magna exceeds 10 mm and is diagnosed and accepted if there is normal vermian morphology and hydrocephalus [10,11].

Statistical analysis

Statistical analyses were performed using IBM SPSS Statistics for Windows, Version 23 (Released 2016; IBM Corp., Armonk, New York, United States). Continuous variables were assessed for normality (Kolmogorov-Smirnov test) and presented as mean ± standard deviation (SD) or median (interquartile range (IQR)). Categorical variables were presented as frequencies and percentages (%). During group comparisons, the Mann-Whitney U test was used for continuous variables, and the chi-square test was used for categorical variables. A p-value < 0.05 was considered statistically significant.

For multivariable logistic regression models, two regression models were created, namely the headache model (outcome = presence of headache) and the VBI model (outcome = presence of VBI symptoms). Predictors included as QAC AP diameter, EI, quadrigeminal cistern diameter, right and left vertebral artery diameters, age, sex, and presence of MCM. Results were reported as odds ratios (ORs) with 95% confidence intervals (CIs). Model fit was evaluated using the Hosmer-Lemeshow test.

## Results

A total of 73 patients with QACs were included (36 females (49.3%), 37 males (50.7%); mean age 52.4 ± 19.0 years; range, 14-95 years). Baseline demographic, clinical, and imaging characteristics stratified by sex are presented in Table 1.

**Table 1:** Baseline demographic, clinical, and imaging characteristics. Values are presented as mean ± standard deviation (SD) and n (%), as appropriate. VBI: vertebrobasilar insufficiency

Variable	Female (n = 36)	Male (n = 37)	Total (n = 73)
Age, years (mean ± SD)	52.1 ± 19.5	52.7 ± 18.7	52.4 ± 19.0
Quadrigeminal cyst AP diameter, mm (mean ± SD)	16.2 ± 4.2	17.7 ± 5.3	16.9 ± 4.8
Quadrigeminal cistern diameter, mm (mean ± SD)	22.6 ± 4.4	23.6 ± 5.8	23.1 ± 5.2
Evans Index (mean ± SD)	0.25 ± 0.42	0.24 ± 0.04	0.24 ± 0.08
Right vertebral artery diameter, mm (mean ± SD)	2.43 ± 0.91	2.50 ± 0.75	2.46 ± 0.83
Left vertebral artery diameter, mm (mean ± SD)	2.31 ± 0.68	2.42 ± 0.78	2.36 ± 0.73
Headache, n (%)	26 (72.2)	23 (62.2)	49 (67.1)
VBI symptoms, n (%)	11 (30.6)	12 (32.4)	23 (31.5)
Mega cisterna magna, n (%)	7 (19.4)	9 (24.3)	16 (21.9)

Headache was reported in 49 patients (67.1%), and VBI symptoms were present in 23 patients (31.5%). MCM was identified in 16 patients (21.9%). The mean QAC AP diameter was 16.9 ± 4.8 mm, and the mean quadrigeminal cistern diameter was 23.1 ± 5.2 mm. The mean EI was 0.24 ± 0.08. Mean right and left vertebral artery diameters measured at the V4 segment were 2.46 ± 0.83 mm and 2.36 ± 0.73 mm, respectively (Table 1).

Continuous variables were also evaluated using the Mann-Whitney U test (Table 2), and no statistically significant difference was found for any value (p > 0.05). The relationship between categorical variables such as sex (female/male), headache (+), and VBI symptoms (+) was also evaluated using the chi-square test. Accordingly, no statistically significant relationship (p > 0.05) was found between sex and headache (χ^2^: 0.947, p-value: 0.619), sex and VBI (χ^2^: 0.692, p-value: 0.486), and headache and VBI (χ^2^: 0.797, p-value: 0.539).

**Table 2:** Comparison of continuous variables. Values are presented as mean ± standard deviation (SD) and n (%), as appropriate. AP: anteroposterior

Variable	Female (n = 36)	Male (n = 37)	Mann-Whitney U test (p-value)
Age, years (mean ± SD)	52.1 ± 19.5	52.7 ± 18.7	p > 0.05 (0.645)
Quadrigeminal cyst AP diameter, mm (mean ± SD)	16.2 ± 4.2	17.7 ± 5.3	p > 0.05 (0.187)
Quadrigeminal cistern diameter, mm (mean ± SD)	22.6 ± 4.4	23.6 ± 5.8	p > 0.05 (0.291)
Evans Index (mean ± SD)	0.25 ± 0.42	0.24 ± 0.04	p > 0.05 (0.839)
Right vertebral artery diameter, mm (mean ± SD)	2.43 ± 0.91	2.50 ± 0.75	p > 0.05 (0.311)
Left vertebral artery diameter, mm (mean ± SD)	2.31 ± 0.68	2.42 ± 0.78	p > 0.05 (0.274)

Multivariable logistic regression analysis demonstrated that none of the imaging or demographic variables independently predicted the presence of headache (Table 3).

**Table 3:** Logistic regression analysis for predictors of headache and vertebrobasilar insufficiency (VBI) Odds ratios (ORs) and 95% confidence intervals (CIs) were estimated using multivariable logistic regression. Statistical significance codes: *** p < 0.001. AP: anteroposterior

Predictor	Headache OR (95% CI)	p-value	VBI OR (95% CI)	p-value
Quadrigeminal cyst AP diameter	1.12 (0.95-1.32)	0.16	1.01 (0.84-1.23)	0.88
Evans Index	0.00 (0.00-613.7)	0.26	0.73 (0.06-8.74)	0.81
Quadrigeminal cistern diameter	0.91 (0.78-1.05)	0.19	0.84 (0.70-1.02)	0.08
Right vertebral artery diameter	1.10 (0.64-1.90)	0.72	0.56 (0.29-1.07)	0.08
Left vertebral artery diameter	1.58 (0.90-2.79)	0.11	0.32 (0.16-0.64)	0.001***
Sex (female, male)	2.26 (0.72-7.11)	0.16	0.40 (0.11-1.46)	0.16
Age	1.00 (0.96-1.03)	0.80	1.03 (0.99-1.06)	0.14
Mega cisterna magna	2.49 (0.53-11.8)	0.25	1.73 (0.33-8.98)	0.52

A larger QAC AP diameter showed a non-significant trend toward association with headache (OR 1.12, 95% CI 0.95-1.32; p-value = 0.16). Left vertebral artery diameter demonstrated a weak, non-significant tendency toward association (OR 1.58, 95% CI 0.90-2.79; p-value = 0.11). Quadrigeminal cistern diameter showed a mild, non-significant negative trend (OR 0.91, 95% CI 0.78-1.05; p-value = 0.19). Age, sex, EI, and the presence of MCM were not significant predictors (p > 0.05). Overall, imaging characteristics did not contribute meaningfully to the likelihood of headache in patients with QAC (Table 3).

In contrast to the headache model, the VBI model identified a significant vascular predictor. A smaller left vertebral artery diameter was strongly associated with the presence of VBI symptoms (OR 0.32, 95% CI 0.16-0.64; p-value = 0.001). Borderline associations were observed for smaller quadrigeminal cistern diameter (OR 0.84, 95% CI 0.70-1.02; p-value = 0.08) and smaller right vertebral artery diameter (OR 0.56, 95% CI 0.29-1.07; p-value = 0.08). QAC AP diameter, EI, sex, age, and presence of MCM did not significantly contribute to VBI symptoms(p > 0.05). These findings highlight the relevance of vertebral artery caliber, particularly on the left side, in patients presenting with symptoms consistent with VBI (Table 3).

## Discussion

Headache is among the most frequently reported symptoms in patients with intracranial arachnoid cysts, with rates ranging from 60% to 70% in prior studies [1,2,6-8]. In QACs, headache is traditionally attributed to aqueductal compression or secondary hydrocephalus [4,5]. Although headache was common in our cohort (67.1%), no radiologic parameter, including QAC AP diameter, quadrigeminal cistern width, or EI, independently predicted its presence.

This finding is consistent with prior observations that headache in arachnoid cysts is often multifactorial [3,6]. Proposed mechanisms include subtle alterations in CSF flow, intermittent venous compression in the posterior fossa, distortion of pain-sensitive structures, or coexistence of primary headache disorders unrelated to the cyst. Importantly, earlier reports have shown that headache does not reliably correlate with cyst size and that surgical decompression may not consistently alleviate symptoms [7,8]. Our results reinforce the need for careful clinical evaluation and cautious patient selection when considering invasive treatment for QAC-associated headache.

The most notable finding of this study was the strong association between a smaller left vertebral artery diameter and VBI symptoms. VAH is a common anatomic variant with a prevalence of 10-25% in the general population [11,12]. It has been increasingly linked to posterior circulation ischemia, isolated vertigo, and transient neurological symptoms [13-15]. Reduced flow reserve in hypoplastic arteries may predispose patients to symptoms even in the absence of major infarction [16,17].

Our results align with these prior observations by demonstrating that reduced left vertebral artery caliber significantly increases the odds of VBI symptoms. The borderline association between smaller right vertebral artery diameter and VBI supports the broader role of vertebral artery asymmetry in posterior circulation hemodynamics [15,16]. Additionally, the trend toward an association between smaller quadrigeminal cistern diameter and VBI is intriguing. A narrower posterior fossa CSF space may limit physiologic buffering capacity or reflect subtle anatomical crowding, potentially amplifying the clinical effect of vertebrobasilar vascular insufficiency. This observation is novel and warrants further investigation.

MCM was present in 21.9% of patients but showed no association with headache or VBI symptoms. MCM is typically regarded as an incidental, benign CSF space variant without clinical consequence [18,19]. Distinguishing MCM from arachnoid cysts remains important because only the latter have the potential to exert mass effect. Consistent with previous studies, our findings suggest that the coexistence of MCM does not influence symptom burden in patients with QAC [20].

Clinical implications

The clinical relevance of our findings is twofold. First, headache in QAC patients appears unlikely to be explained solely by cyst size or ventricular enlargement, reinforcing the need for individualized assessment and avoidance of unnecessary interventions. Second, patients presenting with VBI symptoms may benefit from targeted vascular evaluation, with particular attention to vertebral artery diameters. Identification of VAH may guide further neurologic assessment and influence management decisions, even when the QAC itself appears modest in size.

Limitations

This study has several limitations. The retrospective design introduces potential selection bias and limits control over imaging protocol variability. Vertebral artery measurements were primarily obtained from MRA, which may underestimate vessel caliber compared with CTA or digital subtraction angiography. Although our cohort is larger than most previous QAC series, the sample size may still be insufficient to detect more subtle associations in multivariable models. Finally, detailed hemodynamic data such as flow quantification or perfusion metrics were not available, which may have provided deeper insight into the mechanisms underlying VBI symptoms.

## Conclusions

In patients with QACs, VBI was strongly associated with smaller vertebral artery diameters, particularly on the left side, whereas headache showed no independent radiologic predictors. These findings suggest that vascular factors, rather than cyst dimensions or ventricular measurements, may play a predominant role in posterior circulation symptoms. Incorporating targeted vascular evaluation into the assessment of symptomatic patients may therefore be clinically valuable. Future prospective studies incorporating hemodynamic measurements will be essential to further elucidate the interaction between cyst anatomy, posterior fossa CSF dynamics, and vertebrobasilar vascular variants.
